# 3D Evaluation of Maxillary Sinus Using Computed Tomography: A Sexual Dimorphic Study

**DOI:** 10.1155/2017/9017078

**Published:** 2017-04-04

**Authors:** Balaji Babu Bangi, Uday Ginjupally, Lakshmi Kavitha Nadendla, Bhavana Vadla

**Affiliations:** Department of Oral Medicine and Radiology, Kamineni Institute of Dental Sciences, Narketpally, Nalgonda, Telangana, India

## Abstract

*Purpose*. Gender determination is considered to be an important step in the reconstruction of the biological profile in forensic medicine. Maxillary sinus can be used for identification of sex when other methods are indecisive. Computed tomography (CT) provides an excellent method for examining maxillary sinuses. Hence the aim of the study was to determine the accuracy of gender determination using maxillary sinus with CT.* Materials and Methods*. CT images were used to measure the mediolateral, superoinferior, and anteroposterior dimensions and the volume of the maxillary sinuses in 100 patients (50 males and 50 females) to determine the gender of an individual for forensic identification. Discriminative analysis was done using the values derived and the *t*-test for independent samples was used to compare these values in males and females.* Results*. The accuracy rate was found to be 84% in males and 92% in females with the mean accuracy of 88%.* Conclusion*. Our study concludes that gender determination can be done using measurements of maxillary sinus through CT when other methods are unavailable. The prediction rate can be increased by including volume of the maxillary sinus.

## 1. Introduction

Forensic science is the application of science to criminal and civil laws. Forensic identification of a human body or remains will be relied on by the prosecution in a criminal trial. Gender and age estimations are challenging and important procedures in the identification of unknown skull. Gender determination is part of forensic odontology and it is very important especially when natural calamities, mass disasters, and crime occur where forensic investigator receives unknown skeletal remains [1–3].

Radiological identification of gender is employed when body is decomposed and fragmented. Various body parts are being used for gender determination such as skull, pelvis, long bones, foramen magnum, sella turcica, mandibular ramus, and paranasal sinuses in unknown remains. But in many instances these bones were recovered either in a fragmented or incomplete state where gender determination is difficult to perform. So, it is key to use denser bones that are often recovered intact such as maxillary sinuses [4–6].

The maxillary sinus is the largest of paranasal sinuses located in the body of maxilla. The sinus develops at 10 weeks of intrauterine life and it is first to develop. It opens in the middle nasal meatus of the nasal cavity. At birth size is of pea and enlarges after eruption of deciduous teeth and again, with permanent teeth and with the completion of the eruption of the third molars, the pneumatization of the sinus ends. Szilvassy suggested various shapes of maxillary sinus, that is, triangular, leaf, scapular, and renal shaped. It has been reported that triangular sinuses were the most common in both females and males [7]. It varies greatly in size, shape, and position not only in different individuals but also in different sides of the same individuals. Hence maxillary sinus can be used for gender determination.

Sinus radiography has been used for identification of remains and determination of gender. It has been reported that CT scans are excellent imaging modality in the identification of unknown remains as they provide an accurate assessment of the paranasal sinuses and craniofacial bones [8]. CT measurements of maxillary sinuses, that is, length, width, height, and volume, may be useful for supporting gender determination in forensic medicine.

The aim of the study was to determine the accuracy of gender using maxillary sinus measurements [mediolateral (ML), superoinferior (SI), and anteroposterior (AP) linear dimensions and volume of the maxillary sinuses] through CT scanning.

## 2. Materials and Methods

A retrospective study comprising 100 subjects (50 males and 50 females) above the age group of 20 years is included in the study. Subjects with fracture of maxillary sinus, congenital developmental abnormalities, sinusitis, and other sinus pathologies were excluded from the study. The dimensions of right and left maxillary sinuses of 100 subjects from plain CT were measured using SYNGO software.

**Figure 1:**
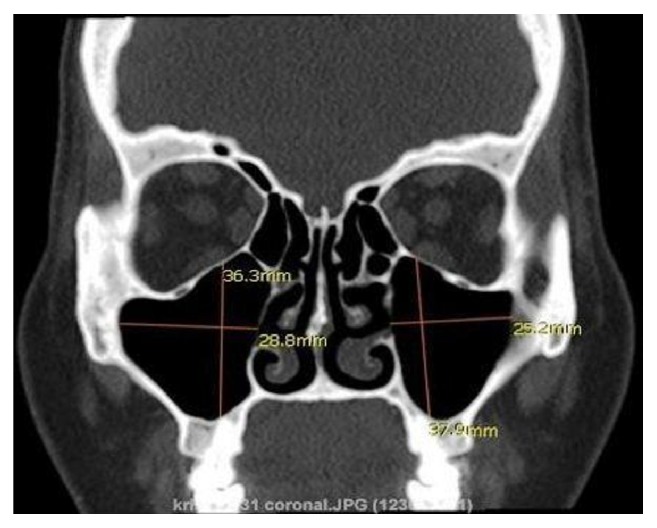
Linear measurements of mediolateral and superoinferior dimensions of maxillary sinus.

**Figure 2:**
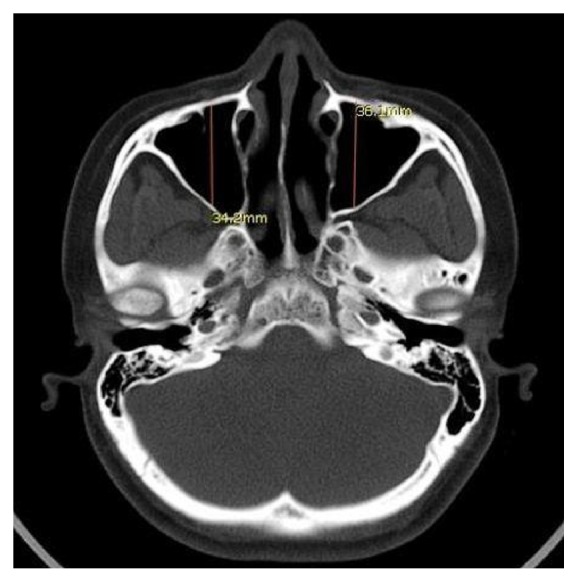
Linear measurements of anteroposterior dimensions of maxillary sinus.

Linear measurements [ML, SI, and AP] of right and left maxillary sinus were made with the help of the CT workstation where the maxillary sinus was in its widest position. ML (width) and SI (height) measurement were performed on coronal images (Figure 1), whereas measurements of AP (depth) were performed on axial images (Figure 2) where the maxillary sinus was in the widest position. Maxillary sinus volume (*V*) was calculated using following equation: Volume = (height × depth × width) × 0.5.

The *t*-test for independent samples was used to compare these values in two groups. A discriminative analysis was performed among all maxillary sinus measurements to deflect gender. The analysis was performed using SPSS version 19.0.

## 3. Results

The mean values for the linear measurements of the right and left maxillary sinuses were calculated (Table 1).

For the right side maxillary sinus, the mean value of ML, SI, and AP for males is 3.30 ± 3.21 cm, 3.16 ± 0.51 cm, and 3.57 ± 0.41 cm, respectively, and in case of females it was 2.48 ± 0.44, 2.92 ± 0.53, and 3.37 ± 0.41, respectively.

For the left side maxillary sinus, the mean value of ML, SI, and AP for males is 2.61 ± 0.54, 3.17 ± 0.5, and 3.55 ± 0.38, respectively, and in case of females it was 2.44 ± 0.42, 2.93 ± 0.54, and 3.38 ± 0.38, respectively, which showed statistically significant larger dimensions in males when compared to females.

The mean values of right and left maxillary sinus volumes for both males and females were calculated (Table 2).

The mean volume of right maxillary sinus in males is 15.23 ± 6.17 whereas in females it is 15.38 ± 6.1. For left maxillary sinus, the mean value in males is 13.35 ± 6.1 and for females it is 12.77 ± 5.49. Volume of left maxillary sinus of males is larger than females.

A discriminative analysis was performed among all sinuses measurements to detect gender. For gender discrimination from measurements of right maxillary sinus the following formula could be used.

(1) Gender=−11.919+0.204×MLR+1.906×SIR+2.330×APR−0.180×VR.

For gender determination from measurements of left Maxillary sinus the following formula could be used:

(2) Gender=−8.552+0.347×MLL+1.020×SIL+1.376×APL−0.015×VL.

By combining right and left maxillary sinus dimensions the following formula could to be used:

(3) Gender=−11.257+0.172×MLR+3.485×SIR+3.353×APR−0.510×VR+0.522×MLL−1.847×SIL−1.460×APL+0.366×VL.

When the variables in the formulae derived above are substituted with the derived values, a numeric value is obtained for the gender. When this value is positive (+Ve) the gender is predicted to be male. A negative (−Ve) value is predicted to be female.

The accuracy of gender predicted was found to be 72% in both males and females from measurements of right maxillary sinus. The accuracy of gender predicted was found to be 72% in males and 76% in females from measurements of left maxillary sinus. The accuracy of gender predicted was found to be 84% in males and 92% in females by combining right and left maxillary sinus dimensions.

## 4. Discussion

Identification of skeletal and decomposed human remains is one of the most difficult skills in forensic medicine. Gender determination is a subdivision of forensic medicine and it is very important especially when information relating to the deceased is unavailable. Gender determination can be done with 100% accuracy when skeleton exists completely. In existence of pelvis and cranium accuracy rate is 98%, 95% with only pelvis and long bones and 80–90% with only long bones [1]. In a study on the subject of gender determination, the accuracy rate of the thickness of the skull was found to be 74.7% in males and 67.6% in females [9]. The accuracy rate using circumference and area of the foramen magnum was 67.0% and 69.3%, respectively [10]. Nascimento Correia Lima et al., in their study of the linear measurement of palatal bone and skull base showed significant sexual dimorphism, with reliability rates of 63.0% and 65.0% [11].

Skull is the most reliable method until after puberty when pelvis is unavailable. CT scan is available in most hospitals as it is indicated in various medical and dental reasons. Teke et al. had reported on accuracy rate of 69.4% in females and 69.2% in males and mean accuracy of 69.3% using only length, width, and height measurements of maxillary sinus [1]. Uthman et al. had reported on accuracy rate of 74.4% in males and 73.3% in females using reconstructed helical CT images of maxillary sinus [6]. Ekizoglu et al. had conducted a morphometric analysis of maxillary sinuses using 1 mm slice thickness multidetector CT to determine the gender and accuracy rate was detected as 80% for women and 74.3% for men with an overall rate of 77.15% [12].

Sahlstrand-Johnson et al. reported that the volume of the maxillary sinus can also be accurately estimated by using a simple formula: (ML dimension × SI dimension × AP dimension) divided by two [13]. Our study includes volume of the maxillary sinus along with three other linear dimensions (ML, SI, and AP) for gender determination. It was observed that if the volume is also included as a variable in the discriminative analysis along with the other three dimensions, the accuracy of gender prediction increases to 84% in males and 92% in females.

It has been reported that the maxillary sinuses are significantly larger in males than in females. Our study measured the ML, SI, and AP dimensions of the maxillary sinus on CT in 100 patients including both males (*n* = 50) and females (*n* = 50). The right maxillary sinus showed an average size of 3.30 × 3.16 × 3.57 cm in males and 2.48 × 2.92 × 3.37 cm in females. The left maxillary sinus showed an average size of 2.61 × 3.17 × 3.55 mm in males and 2.44 × 2.93 × 3.38 mm in females. The right maxillary sinus has been reported to be larger than the left sinus in both genders.

Though CBCT is characterized by a lower effective radiation dosage, lower cost, easy accessibility, and somehow shorter acquisition time compared to CT and MRI, their number is still limited to validate its full potential in the field of forensic science as it has some disadvantages such as low contrast range, limited field of view, limited scanned volume, and not being used for estimation of Hounsfield units [14, 15]. A study by Saccucci et al. had reported that it is not possible to support the use of maxillary sinuses to discern sexual difference in corpse identification using CBCT [16].

## 5. Conclusion

Gender determination plays a major role in forensic medicine. A thorough knowledge and usage of the appropriate evidence from forensic science enables proper identification of the individual. It has been reported that maxillary sinuses remain intact despite the skull and other bones getting badly disfigured in victims who are incinerated. Therefore, maxillary sinuses can be used for identification. Computed tomography (CT) provides an excellent method for examining maxillary sinuses and the machine is available in most of the hospitals. Our study concludes that given a cranium of unknown origin gender determination can be done using maxillary sinus dimensions through computed tomography.

## Figures and Tables

**Table 1:** Distribution of maxillary sinus dimensions measured on CT.

Dimensions	Mean ± SD		*P*	*t*
	Males	Females		
MLR	3.301 ± 3.210	2.485 ± 0.440	*P* > 0.05	1.257
SLR	3.166 ± 0.515	2.922 ± 0.539	*P* > 0.05	1.264
APR	3.576 ± 0.413	3.376 ± 0.413	*P* > 0.05	1.637
MLL	2.618 ± 0.548	2.443 ± 0.423	*P* > 0.05	1.610
SLL	3.170 ± 0.502	2.931 ± 0.545	*P* > 0.05	1.708
APL	3.559 ± 0.385	3.384 ± 0.388	*P* > 0.05	1.064

MLR: mediolateral dimension of right maxillary sinus, SIR: superoinferior dimension of right maxillary sinus, APR: anteroposterior dimension of right maxillary sinus, MLL: mediolateral dimension of left maxillary sinus, SIL: superoinferior dimension of left maxillary sinus, and APL: anteroposterior dimension of left maxillary sinus.

**Table 2:** Distribution of maxillary sinus volume measured on CT.

Dimensions	Mean ± SD		*P*	*t*
	Male	Female		
VR	15.23 ± 6.17	15.38 ± 6.10	*P* > 0.05	1.085
VL	13.35 ± 6.10	12.77 ± 5.49	*P* > 0.05	1.592

VR: volume of right maxillary sinus, VL: volume of left maxillary sinus, and SD: standard deviation.
